# Integrating GWAS with a gene co‐expression network better prioritizes candidate genes associated with root metaxylem phenes in maize

**DOI:** 10.1002/tpg2.20489

**Published:** 2024-07-22

**Authors:** Stephanie P. Klein, Shawn M. Kaeppler, Kathleen M. Brown, Jonathan P. Lynch

**Affiliations:** ^1^ Interdepartmental Graduate Degree Program in Plant Biology The Pennsylvania State University University Park Pennsylvania USA; ^2^ Department of Agronomy University of Wisconsin Madison Wisconsin USA; ^3^ Department of Plant Science The Pennsylvania State University University Park Pennsylvania USA

## Abstract

Root metaxylems are phenotypically diverse structures whose function is particularly important under drought stress. Significant research has dissected the genetic machinery underlying metaxylem phenotypes in dicots, but that of monocots are relatively underexplored. In maize (*Zea mays*), a robust pipeline integrated a genome‐wide association study (GWAS) of root metaxylem phenes under well‐watered and water‐stress conditions with a gene co‐expression network to prioritize the strongest gene candidates. We identified 244 candidate genes by GWAS, of which 103 reside in gene co‐expression modules most relevant to xylem development. Several candidate genes may be involved in biosynthetic processes related to the cell wall, hormone signaling, oxidative stress responses, and drought responses. Of those, six gene candidates were detected in multiple root metaxylem phenes in both well‐watered and water‐stress conditions. We posit that candidate genes that are more essential to network function based on gene co‐expression (i.e., hubs or bottlenecks) should be prioritized and classify 33 essential genes for further investigation. Our study demonstrates a new strategy for identifying promising gene candidates and presents several gene candidates that may enhance our understanding of vascular development and responses to drought in cereals.

## Core Ideas

- Root metaxylem phenotypes are an under‐explored target for crop improvement for drought resilience.
- A traditional genome‐wide association study (GWAS) paired with a gene co‐expression network found candidate genes underlying root metaxylem phenes.
- The best candidates are likely those with relevant functional roles and genes “essential” to network function.

**Abbreviations**
ABA: abscisic acid
BN: bottleneck gene
GWAS: genome‐wide association study
HUB: hub gene
SNP: single nucleotide polymorphism
WS: water stress
WW: well watered

## 1 INTRODUCTION

Drought is a primary constraint to global crop production and its effects are predicted to worsen in the coming decades as a result of climate change (Dai, 2013; IPCC, 2013; Lobell et al., 2011). Plant breeding efforts have been aimed at maintaining crop productivity in stressful environments to sustain growing agriculture‐centric economies and meet increasing consumer demand. Maize (*Zea mays*), one of the world's most economically and nutritionally important crops, is particularly sensitive to drought stress (Daryanto et al., 2016; Lobell et al., 2014). As the primary organ responsible for the sensing and uptake of water, roots have become a focus for crop improvement under drought (Lynch et al., 2014; Vadez et al., 2014). Recent efforts have sought to approach root‐focused breeding by selecting for a root system architecture better adapted to water‐limited conditions (Burridge et al., 2020; Ho et al., 2005; Lynch, 2013, 2022; Lynch et al., 2021; Tardieu et al., 2017; Zhang et al., 2019). For instance, a steep root growth angle that increases rooting depth has been associated with improved tolerance to water scarcity in maize (Liakat Ali et al., 2015; Pires et al., 2020) and rice (Uga et al., 2013). Fewer nodal roots with more sparsely distributed long lateral roots are also associated with improved drought tolerance (Gao & Lynch, 2016; Lynch, 2019; Zhan et al., 2015). Several anatomical phenes (phene is to phenotype as gene is to genotype; sensu [York et al., 2013]) have been proposed to mitigate drought effects by facilitating the capture of mobile resources, such as water and nitrogen, deep in the soil profile (Lynch, 2013, 2018, 2019; Lynch et al., 2014, 2021). Increased aerenchyma production (Chimungu et al., 2015; Jaramillo et al., 2013; Zhu et al., 2010) and larger cortical cells arranged in fewer files cheapen the root maintenance costs (Chimungu et al., 2014a, 2014b), which would allow more resources to be reallocated to the construction of deeper roots (Lynch, 2013; Lynch et al., 2014). At the cellular level, modifying the activity of plasma membrane intrinsic proteins, like *PIP2;5*, has been demonstrated to improve tolerance to mild drought stress (Ding et al., 2020). Despite a rich history of research and having direct implications for water uptake and transport (Maurel & Nacry, 2020; Vadez, 2014; Wasson et al., 2012), root hydraulics have been an underutilized target for crop improvement.

Root metaxylem vessels are a natural target to improve maize since their size and number in the root are related to abiotic stress tolerance (Comas et al., 2013; Richards & Passioura, 1989). Several studies have found an association between drought tolerance and metaxylem vessel number (de Souza et al., 2013; Klein et al., 2020) or metaxylem vessel area (Abd Allah et al., 2010; de Souza et al., 2013; Klein et al., 2020; Purushothaman et al., 2013; Richards & Passioura, 1989). Plants with narrower root metaxylem have reduced hydraulic conductivity but more conservative water usage and greater resistance to cavitation (Guet et al., 2015; Richards & Passioura, 1989; Sperry & Saliendra, 1994; Vilagrosa et al., 2012). Given that hydraulic conductance is proportional to the vessel radius raised to the fourth power according to the Hagen‐Poiseuille equation, even a slight narrowing of the metaxylem vessels will result in a considerable reduction in axial conductivity. Conservative water usage because of restricted hydraulic conductance, especially early in the growing season, has been suggested to be more effective than deeper rooting by making water access more likely during the critical anthesis‐silking interval (Feng et al., 2016; Zaman‐Allah et al., 2011). Reduced transpiration due to restricted hydraulic conductance from narrow metaxylem vessels may also be beneficial for mitigating drought effects by inducing stomatal closure, increasing water use efficiency by reducing the overall shoot size, and retaining moisture at the root tips to enable further growth through the soil profile (Lynch, 2019; Tardieu et al., 2017; Vadez et al., 2014). A constitutive reduction in metaxylem vessel area could limit plant maximum potential growth or reduce relative growth rates under well‐watered (WW) conditions (Comas et al., 2013; Wahl & Ryser, 2000), but these effects may be counteracted by augmenting the number of metaxylem vessels (Lynch et al., 2014). Metaxylem phenes that improve performance under water stress (WS) with no penalty under optimal conditions could be valuable in efforts to develop more climate‐resilient crops.

Metaxylem vessels form through the coordination of phytohormone biosynthesis and signaling, cell wall formation and lignification, cell expansion, radial patterning, and programmed cell death (Milhinhos & Miguel, 2013; Roberts & McCann, 2000; Růžička et al., 2015; Schuetz et al., 2012). Decades of research have deepened our understanding of the genetic and developmental underpinnings of xylem development, but these efforts have mostly been limited to dicotyledonous or woody species giving us a limited view on genetic components uniquely operating in monocot species. Recent studies have identified quantitative trait loci associated with xylem area in spring barley (*Hordeum vulgare*) (Oyiga et al., 2020) and metaxylem size and number in rice (*Oryza sativa*) (Fonta et al., 2023; Kadam et al., 2017). However, the genetic mechanisms regulating xylem development in grain crops are still poorly understood. Arranged in a polyarchy pattern (Hochholdinger, 2009), the vascular organization of monocot roots, such as members of the *Poaceae*, is structurally distinct from di‐, tri‐, or tetrarch patterns typical of most dicots. Cell wall composition also differs between dicots and monocots, since monocots are richer in hydroxycinnamates and hemicellulose with little pectin or structural protein content (Carpita, 1996; Smith & Harris, 1999; Vogel, 2008). Despite these differences, shared genetic machinery underlying xylem development is likely. Comparisons between maize and closely related grasses have found shared genetic architecture influencing sorghum (*Sorghum bicolor*) root architecture (Zheng et al., 2020) and rice grain development (Chen et al., 2016). Identifying unique genetic components regulating monocot vascular development can be challenging because inferred gene function in monocots relies on functional annotation derived from dicot model species (e.g., *Arabidopsis thaliana*). Nevertheless, dicot‐based annotations are invaluable for identifying candidate genes with conserved functional roles. Individual genetic components are shared among much of plant life, like the cellulose synthase A (*CesA*) gene family in all seed plants (Richmond & Somerville, 2000). However, the mechanisms contributing to monocot xylem development are poorly understood overall, and at this point, it is difficult to discern the genetic architecture that is conserved from that which is unique to monocots.

Plasticity in xylem phenotypes in response to drought has been observed in maize (Klein et al., 2020; Schneider, Klein, Hanlon, Kaeppler, et al., 2020), rice (Henry et al., 2012; Kadam et al., 2017), and wheat (*Triticum aestivum*) (Kadam et al., 2015), and is likely mediated by stress‐responsive cellular and genetic factors. The genetic mechanisms underlying drought‐induced variation in root xylem phenotypes are largely unknown. Several genes regulating the biosynthesis and signaling of brassinosteroids, cytokinins, ethylene, and gibberellin have been directly implicated in constitutive and abiotic stress‐responsive xylem development in dicot roots (Milhinhos & Miguel, 2013; Ramachandran et al., 2020). However, these complex signaling cascades facilitated by hormones are likely modified by crosstalk with other phytohormones like abscisic acid (ABA), whose production increases in response to drought (Finkelstein, 2013; Ramachandran et al., 2020). In maize, increased ABA production induced by drought coincides with increased root hydraulic conductivity, which is likely mediated by the augmented activity of root aquaporins rather than changes to root metaxylem anatomy (Hose et al., 2000; Parent et al., 2009). More recent work showed that ABA interfered with signaling among key developmental regulators and subsequently altered root xylem phenotypes in *Arabidopsis* under limited water availability (Ramachandran et al., 2018). However, there are likely many more genetic components participating in these signaling cascades that contribute to variation in xylem phenotypes and water uptake.

**Core Ideas**

- Root metaxylem phenotypes are an under‐explored target for crop improvement for drought resilience.
- A traditional genome‐wide association study (GWAS) paired with a gene co‐expression network found candidate genes underlying root metaxylem phenes.
- The best candidates are likely those with relevant functional roles and genes “essential” to network function.

Novel breeding and phenotyping technologies have increased the capacity to evaluate and map desirable characteristics to genetic loci in large populations, as is done in a genome‐wide association study (GWAS). Even so, many challenges remain when analyzing GWAS outputs to avoid false positives and isolate the true loci with significant causal effects on phenotype. One method is to couple GWAS with a gene co‐expression network, which is a helpful tool to infer a candidate gene's biological function relative to the functional role filled by comparably expressed genes (He et al., 2023; Schaefer et al., 2018, 2017). Complex quantitative traits are often pleiotropic and are associated with many significant single nucleotide polymorphisms (SNPs) with small effect sizes (Ingvarsson & Street, 2011). A candidate gene's topological characteristics within the gene network may provide insight on its biological role and essentiality. For instance, hub genes (HUB) (genes that interact with a large number of other members of the subnetwork) and bottleneck genes (BN) (genes through which many interactions are funneled) are posited to be the most essential for proper function (Jeong et al., 2001; Yu et al., 2004, 2007). Modifying or removing the activity of hub and BN has the greatest chance of inducing measurable downstream effects. Thus, integrating a GWAS with a gene co‐expression network is a powerful method to identify candidate genes with the highest likelihood of shaping a measurable effect on phenotype and function.

Root metaxylem phenotypes merit greater attention because of their potential to improve crop performance under water‐limited conditions. One course of action is to dissect their genetic architecture to provide insight on the loci and overarching mechanisms contributing to root metaxylem phenotypic variation under abiotic stress. In this study, we phenotyped the root metaxylem of a large diversity panel of field‐grown maize under WW and WS conditions. Our objectives were to test the hypotheses that (1) natural variation in root metaxylem phenes is under genetic control; (2) genetic loci associated with root metaxylem phenes under WS are unique from those activated under WW; and (3) integration of gene co‐expression networks with GWAS enables better candidate gene prioritization.

## 2 MATERIALS AND METHODS

### 2.1 Plant materials, field conditions, and experimental design

Subsets of maize genotypes from the Wisconsin Diversity Panel (WiDiv) (Hansey et al., 2011; Hirsch et al., 2014; Mazaheri et al., 2019) were evaluated in the field—176 in 2015 and 412 in 2016—under WW and WS conditions (Table S1). The Wisconsin Diversity Panel is a large collection of inbred maize lines of similar vigor representing the genetic diversity of North American temperate maize. A subset of 95 genotypes were grown in both field seasons. All experiments were conducted at the Apache Root Biology Center located near Willcox, AZ (32°153′9.252″ N, 109°49′56.928″ W). Field trials were conducted in Grabe loam soil (coarse‐loamy, mixed, thermic typic Torrifluvent) from May through October in 2015 and 2016. Each genotype was planted in a single‐row plot of 20 individuals per plot with 23 cm spacing between individuals. Rows were spaced 75 cm apart (5.74 plants m^−2^). Two replications of the WiDiv were grown in each treatment in a randomized complete block design. Experiments were irrigated using a center pivot irrigation system. WW and WS treatments were imposed in separate blocks. For our WW treatment, we calculated the crop water requirements to support optimal plant growth in semiarid environments (Allen et al., 1992). The WS treatment was initiated 4 weeks after planting by withholding 50% of the water needed to sustain adequate growth in the WW blocks. This corresponded to the approximate emergence of the fourth whorl crown roots. The severity of WS was monitored over the course of the season using PR2 multi‐depth soil moisture probes (Dynamax). Prior to planting, fertilizer and lime applications were adjusted according to soil test results to meet the nutritional demands for maize production. Pest and pathogen control were applied across the whole field as needed.

### 2.2 Field phenotyping

Maize root metaxylem phenotypes were evaluated using the shovelomics method (Trachsel et al., 2010) coupled with laser ablation tomography (LAT) (Hall et al., 2019; Strock et al., 2019). To summarize, the root crown of one representative maize plant was excavated from each plot just prior to anthesis in a soil monolith using a standard spade approximately 30 cm wide and deep and washed to remove the remaining soil. Samples to assess the root anatomy were collected by excising two segments from the fourth whorl nodal root, 5–8 cm from the base of the root. The samples were preserved in 75% (v/v) ethanol in water and ablated using LAT. During LAT, the root sample was placed on an imaging stage, moved into a 355 nm pulsed laser (Avia 7000), and vaporized in the focal plane of a camera (Canon T3i with a MP‐E 65 mm micro lens) that simultaneously captured an image of the cross‐section. Metaxylem features were quantified in each cross‐section image using MIPAR software (Sosa et al., 2014). Two images were collected from each root segment and averaged to assess the mean fourth nodal root metaxylem phenotype. The theoretical volumetric flow rate was estimated based on the dimensions and number of metaxylem vessels present in each image using the Exact equations (Berker, 1963; Lewis & Boose, 1995), which estimate the volumetric movement of water through noncircular conduits.

Broad‐sense heritability and repeatability on an entry mean basis of each metaxylem phene were estimated according to Fehr (1991). Marker‐based heritability was calculated using the “heritability” v.1.3 R package (Kruijer et al., 2015). Broad‐sense heritability was estimated with the 95 genotypes grown in both field seasons where the water treatment was included as a cofactor. The subsets of 176 and 412 genotypes grown in 2015 and 2016, respectively, were used to estimate repeatability in each field season.

### 2.3 Assembly of a root gene co‐expression network

Co‐expression analysis and module identification were conducted on a publicly available RNA‐Seq dataset collected on 19 samples comprising multiple tissues and timepoints (Table S8) (Stelpflug et al., 2016) using the Weight Gene Co‐expression Network Analysis (WGCNA) package (Langfelder & Horvath, 2008). Genes with an FPKM < 1 for all samples were omitted from the WGCNA analysis. Parameters were adjusted so that the soft threshold was selected to produce a >90% model fit. Additionally, the minimum module size was 30 and the branch cut height was set to 0.8. Modules with a high likelihood of being related to xylem development were designated as those displaying high average gene expression in the stele (Stele_3d), differentiation zone (DifferentiationZone_3d), meristematic/elongation zone (Mz.Ez_3d), whole root system (WholeRootSystem_3d) 3 days after sowing (DAS), and zones 1 and 2 of the primary root 7 DAS (TapRoot_Z1_7d, TapRoot_Z2_7d). Gene ontology (GO) enrichment was conducted on each xylem‐related module using AgriGO (Du et al., 2010) to correlate possible biological roles with xylem development.

Network topology metrics were calculated to infer the relationship between each gene and the rest of the network. For each gene, closeness centrality, that is, the inverse of the average shortest path between the gene of interest and all other nodes in the network, and betweenness centrality, that is, the number of shortest paths between all pairs of nodes in the network that pass through the gene of interest, were calculated using “igraph” (Csardi & Nepusz, 2006). Genes in the uppermost quintile of closeness centrality and betweenness centrality were selected as hub and BN, respectively. Genes may be labeled as both a hub and bottleneck. Network interactions were visualized using Cytoscape 3.8.0 (Lopes et al., 2010).

### 2.4 Genome‐wide association analysis

GWAS was performed using the phenotypic values obtained from each season and treatment and a panel of 899,784 SNPs assembled using RNA‐Seq data from whole seedlings of each member of the WiDiv (Mazaheri et al., 2019) implemented in the GAPIT package (Lipka et al., 2012) using a mixed linear model in R. SNPs with minor allele frequencies less than 0.01 were discarded from the panel. Due to differences in the genotype membership between each season, the number of SNPs included in each GWAS differed: 578,279 in 2015 and 574,175 in 2016. The first three principal components were used as covariates to control for population structure. The fit of the model was corroborated with quantile–quantile (*Q*–*Q*) plots (Figure S2). The minor allele effects associated with SNPs observed in the 2016 GWAS were tested using the 2015 GWAS to identify SNPs with consistent minor allele effects in both seasons. Past GWAS for root phenes has struggled to identify significant SNPs above the Bonferroni threshold because many root phenes are pleiotropic and individual genes have small effect sizes (Schneider, Klein, Hanlon, Kaeppler, et al., 2020; Schneider, Klein, Hanlon, Nord, et al., 2020). Therefore, we sought an alternative method that would be more liberal to accommodate these considerations while establishing a clear threshold to discern potential candidacy. The significance threshold was selected using a linear model that predicts a significant *p*‐threshold according to the marker‐based heritability (Kaler & Purcell, 2019). Using the mean marker‐based heritability of all phenes (0.264; Table 1), the *p*‐threshold was predicted to be at −log(*p*) ≤ 3.43. A SNP that satisfied the *p*‐threshold in 2016 and displayed consistent minor allele effects in both seasons was designated as “significant.” Candidate genes corresponding to a significant SNP were detected according to the AGPv4 reference sequence assembly (Jiao et al., 2017). Annotation of candidate genes was obtained from MaizeGDB (Portwood et al., 2018).

**TABLE 1:** Two‐way analysis of variance (ANOVA) of root metaxylem phenes measured across two field seasons under well‐watered (WW) and water‐stress (WS) conditions, and broad‐sense and marker‐based heritability calculated for each phene among 95 genotypes planted in each season.

Phene description	Two‐way ANOVA						Broad‐sense heritability (95 genos)	Marker‐based heritability (95 genos)	Repeatability 2015 (176 genos)	Repeatability 2016 (412 genos)
	Year		Treatment		Year × treatment					
	*F*	*p*	*F*	*p*	*F*	*p*				
Maximum individual metaxylem vessel area (mm^2^)	**8.077**	**0.00456**	3.192	0.0742	3.197	0.074	0.625	0.327	0.652	0.67
Median individual metaxylem vessel area (mm^2^)	**5.769**	**0.0165**	1.234	0.267	1.2364	0.266	0.805	0.361	0.668	0.662
Total metaxylem vessel area (mm^2^)	0.0009	0.976	0.237	0.627	0.2386	0.625	0.646	0.133	0.602	0.466
Metaxylem vessel number	**8.661**	**0.00332**	0.592	0.442	0.5906	0.442	0.672	0.251	0.827	0.502
Volumetric flow rate (mm^3^/s)	**11.314**	**0.000794**	**4.412**	**0.0359**	**4.4109**	**0.036**	0.72	0.249	0.8	0.527

*Note*: Repeatability of each phene was calculated using all genotypes grown in each season. Bold numbers in the two‐way ANOVA indicate significant associations (*p* < 0.05).

### 2.5 Data analysis

All data analyses were performed in R version 4.0.0 (R Core Team, 2020) with graphical illustration using “ggplot2” (Wickham, 2016). All data used for phenotypic analysis and subsequent GWAS are provided in the Supporting Information for 2015 (Table S2) and 2016 (Table S3).

## 3 RESULTS AND DISCUSSION

### 3.1 Natural variation exists for maize root phenotypes in the field

**FIGURE 1:**
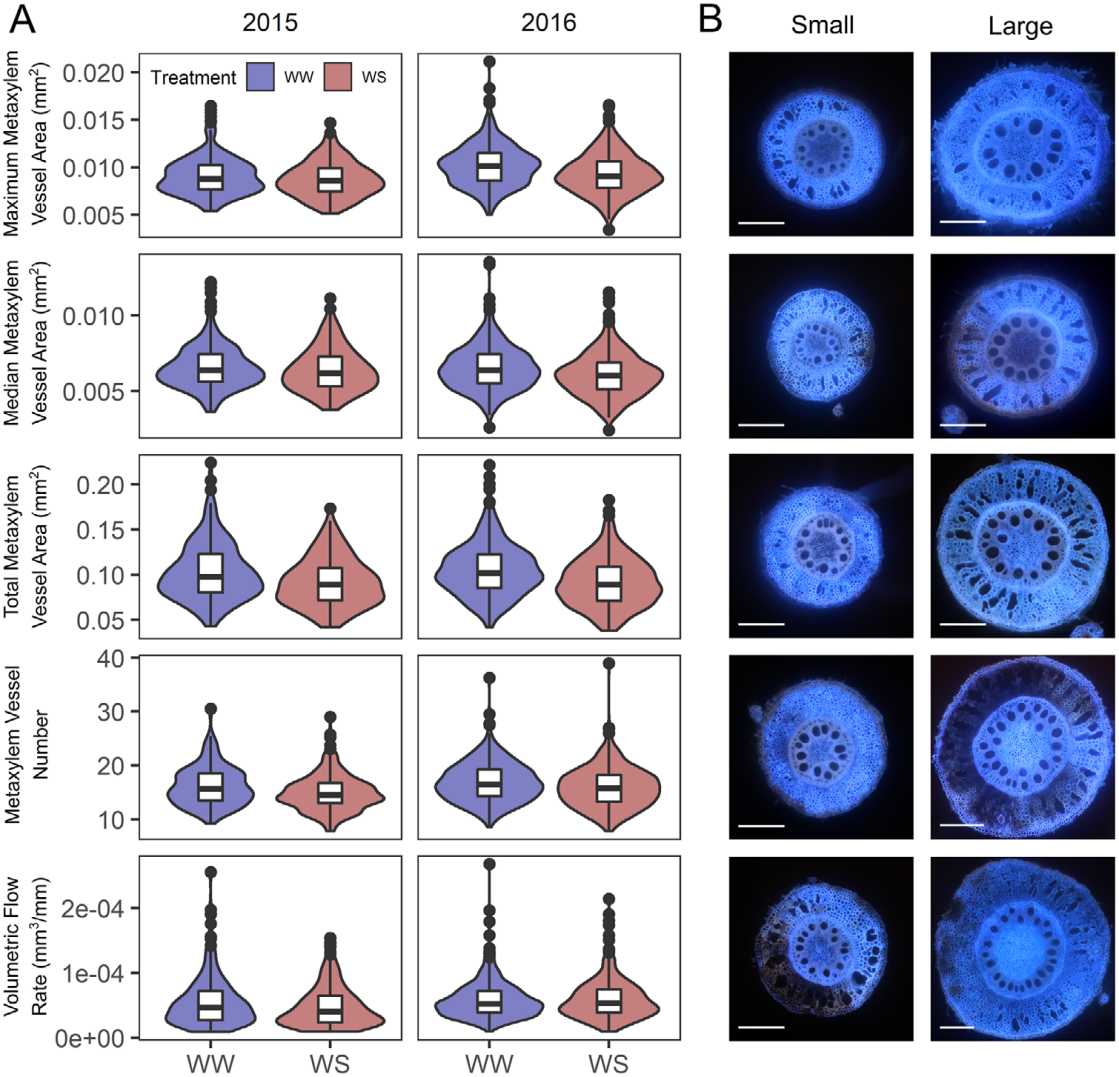
Wide variation exists in root metaxylem phenes under well‐watered (WW) and water‐stress (WS) conditions in the field. (A) Violin plots show the distribution of each root metaxylem phene under WW (blue) and WS (red) in each field season while the overlaid box plots show the median bounded by the first and third quartile. Two‐way analysis of variance (ANOVA) determined statistical difference between treatments and season (Table 1). (B) Root cross‐sectional images collected via laser ablation tomography illustrate the range in each root metaxylem phenotype. White scale bars in the bottom left of each image are equal to 0.5 mm.

Wide variation in all root metaxylem phenes was observed under both WW and drought‐stress (WS) (hereafter, water stress) conditions in 176 and 412 genotypes of the Wisconsin Diversity Panel in 2015 and 2016, respectively (Figure 1). A three‐ to fourfold range in values was observed for all metaxylem phenes under both water treatments except for volumetric flow rate, which exhibited a 31‐fold range. The watering regime had no significant overall effect on metaxylem phenes except for volumetric flow rate (*p* = 0.0359) (Table 1), but responses to WS varied by genotype (Figure S1). Plasticity in all metaxylem vessel phenes in response to WS ranged from −84% to 351% relative to the phene state in WW conditions. While total metaxylem vessel area was stable between seasons, there were significant effects attributed to the growth year for maximum and median metaxylem vessel areas, metaxylem vessel number, and volumetric flow rate (Table 1). Volumetric flow rate was the only phene to exhibit a significant interactive year and treatment effect (*p* = 0.036) (Table 1).

We detected high heritability in root metaxylem phenes (Table 1). Broad‐sense and marker‐based heritability on an entry mean basis was calculated within a shared set of 95 genotypes planted in both seasons. Broad‐sense heritability ranged from 0.625 to 0.805 and marker‐based heritability ranged from 0.133 to 0.361, overall (Table 1). Broad‐sense heritability observed was higher than previously reported values for root anatomical phenes (Kadam et al., 2017; Schneider, Klein, Hanlon, Kaeppler, et al., 2020), but may be slightly conflated given that these values were based on only 95 genotypes that were planted in each season. Repeatability was also calculated for the collection of genotypes grown in each season and ranged from 0.602 to 0.827 in 2015 and 0.466 to 0.670 in 2016 (Table 1).

### 3.2 GWAS detects numerous candidate genes underlying root metaxylem phenes

**FIGURE 2:**
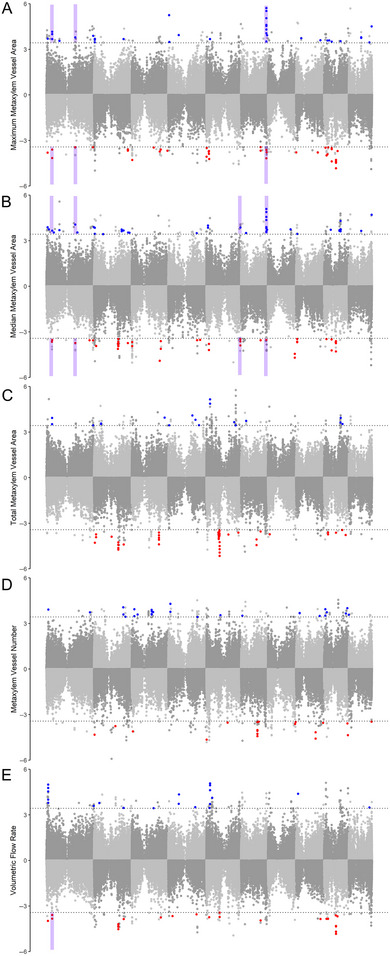
Manhattan plots of the genome‐wide association study (GWAS) results for (A) maximum metaxylem vessel area, (B) median metaxylem vessel area, (C) total metaxylem vessel area, (D) metaxylem vessel number,. and (E) volumetric flow rate in each watering regime in 2016. Results from the well‐watered (WW) studies are shown on the positive axis while the water stress (WS) is shown on the negative axis. The dotted line indicates the significance threshold (−log10(*p*) > 3.43). Dots colored blue and red indicate significant single nucleotide polymorphisms (SNPs) in the WW and WS treatments, respectively, that had consistent allelic effects in the 2015 GWAS. Select genomic regions that shared significant SNPs in both treatments have been highlighted in purple.

Separate GWAS analyses were performed for each treatment and season of field phenotype data to identify genetic loci that consistently exhibited similar effects on variance in root metaxylem phenotypes. Despite differences in taxa membership in each season, there were 550,617 SNPs from the original SNP panel (Mazaheri et al., 2019) included in analyses of each season that were used to evaluate the consistency of minor allele effects on phenotypes (Figure S3A). Few SNPs omitted from the comparison were significant in any analysis. Because of its larger genotype collection, the 2016 GWAS was designated the “pilot set” to identify many genetic loci potentially associated with root metaxylem phenes. The 2015 GWAS was designated the “corroboration set” to corroborate allelic effects observed in the testing set. For the pilot and corroboration sets, the allelic effect of each SNP in each phene was converted to a percentile rank, where 0 denoted a strong negative effect and 100 indicated a strong positive effect on phenotype. A SNP with consistent allelic effects for the same phene was one where the percentile rank in the validation set was within 10 percentile points of its rank in the testing set (Figure S3). This analysis was repeated for each root metaxylem phene and treatment. About 24%–28% of the full SNP panel displayed consistent allelic effects in each GWAS and varied among phenes and treatments (Table S4). A SNP was noted as “significant” if it exceeded the *p*‐threshold and displayed consistent allelic effects on metaxylem phenes. No significant associations were detected above the Bonferroni threshold of −log(*p*) = 9.08. Thus, a more liberal threshold of −log(*p*) = 3.43 was applied to accommodate SNPs associated with small effect sizes (Figure 2). This threshold was estimated using a model that considered the average marker‐based heritability of all root metaxylem phenes and linkage disequilibrium in maize (Kaler & Purcell, 2019).

Overall, 244 gene models (124 in WW and 126 in WS) contained or were within 5 kb of 351 significant SNPs (181 in WW and 176 in WS) associated with maize root metaxylem phenes (Figure 2; Table S5; Table 2). We identified several genomic regions displaying a higher density of significant SNPs associated with a specific phene (Figure 2). For instance, 13 gene models near 25 significant SNPs associated with total metaxylem vessel area under WS were detected in a region on chromosome 5. Seven gene models near 12 significant SNPs associated with maximum and/or median metaxylem vessel area under WW conditions were detected in a region on chromosome 6. Of the candidate genes associated with root metaxylem phenes under WW and WS, approximately 68% were annotated for Mapman ontogenic categories (Figure S4). However, there were few differences in the representation of Mapman ontogenic categories for candidate genes associated with the WW treatment compared to those associated with WS. A slightly larger proportion of candidate genes identified under WS were associated with developmental processes, while a larger proportion of candidate genes identified under WW conditions were associated with stress responses.

**TABLE 2:** Number of significant single nucleotide polymorphisms (SNPs) and candidate genes detected in the maize genome‐wide association study (GWAS) and the number of candidate genes found in one of 14 gene co‐expression modules associated with root tissues most relevant to xylem development.

Phene	Treatment	Number of significant SNPs	Number of gene models within 5 kb of significant SNP	Number of genes in xylem‐related co‐expression modules	Number candidate genes that are essential (BN, HUB, and BN/HUB)
Maximum root metaxylem vessel area	WW	43	26	18	3
	WS	53	43	19	4
Median root metaxylem vessel area	WW	70	44	13	3
	WS	60	29	21	6
Total root metaxylem vessel area	WW	21	20	8	3
	WS	59	35	11	6
Metaxylem vessel number	WW	51	29	16	6
	WS	29	22	9	3
Volumetric flow rate	WW	29	22	10	2
	WS	39	22	5	2
Total	WW	181	124	57	15
	WS	176	126	50	18
	Total	351	244	103	33

Abbreviations: BN, bottleneck gene; HUB, hub gene; WS, water stress; WW, well watered.

We identified several genetic loci that were directly or indirectly associated with drought responses. One candidate gene (Zm00001d038999) encodes the protein drought‐induced 19 homolog7 (*DIN7*) (Table S5), which is a transcriptional activator whose knockout mutant resulted in longer root systems and enhanced tolerance to WS and high salinity stress in *Arabidopsis* (Qin et al., 2014). Interestingly, drought‐induced 19 potentially interacts with *ERD15* (Zm00001d048165) (Qin et al., 2014), which was detected as another candidate gene in our analysis (Table S5). *ERD15* is rapidly activated under WS, is a negative regulator of ABA (Kariola et al., 2006), and was found to mediate osmotic stress‐induced programmed cell death in soybean (*Glycine max*) protoplasts (Alves et al., 2011). Programmed cell death elicits xylem proliferation, so it is particularly intriguing that *ERD15* is associated with metaxylem vessel number in maize. Similarly, the activity of root‐specific kinase 1 (Zm00001d014243), which was associated with volumetric flow rate under WW conditions in maize (Table S5), is induced by dehydration and salt (Hwang & Goodman, 1995). Remarkably, *ERD15* and root‐specific kinase 1 (Zm00001d014243) were identified in a GWAS conducted in rice under no stress as gene candidates associated with metaxylem vessel number and median metaxylem vessel area, respectively (Fonta et al., 2023). Identifying pairs of homologous genes that were similarly detected in separate GWAS's, such as these here, may be another strategy for narrowing the pool of candidate genes.

### 3.3 Distinct genetic architectures likely underpin root metaxylem phenes in each environment

Our initial attempts for candidate prioritization focused on identifying genes associated with SNPs that were similarly significant in analyses conducted in both watering regimes. We found only 13 significant SNPs associated with six candidate genes detected in both watering regimes (Figure 2; Table 3). Several of these candidate genes were also detected in more than one metaxylem phene either by association with a single co‐localized SNP or because multiple SNPs residing near the gene were detected. Of these candidate genes, only one was annotated—Zm00001d018394, DNA‐binding protein phosphatase 2C (*DBPTF2*), which was associated with median root metaxylem vessel area in both water treatments. The other five genes are not annotated, but their functional annotation may be inferred according to their homology with genes characterized in *Arabidopsis* (Figure 2; Table 3). Zm00001d028486 encodes a chemocyanin and contains two SNPs associated with median and maximum root metaxylem vessel areas in both treatments and volumetric flow rate under WS. Zm00001d031332 encodes a heat shock protein and contains four SNPs associated with median and maximum root metaxylem vessel areas under WW and WS conditions. Two SNPs nearby, Zm00001d038573, a ubiquitin domain containing 1, and Zm00001d038754, an endoplasmic reticulum lumen protein‐retaining receptor, were associated with maximum and median root metaxylem vessel area in both treatments. Finally, Zm00001d045954 encodes an RNA‐binding protein that contains one SNP significantly associated with maximum root metaxylem vessel area in both water treatments. Mutation of this gene's ortholog in *Arabidopsis (TOUGH)* has been shown to dramatically impair plant development and reduce vascularization (Calderon‐Villalobos et al., 2005). Because there are few genetic loci associated with root metaxylem phenes that were detected in both water regimes, this suggests that drought induces changes in vascular patterning distinctly different from the processes employed when water is more readily available.

**TABLE 3:** The six candidate genes that were associated with root metaxylem phenes in both well‐watered (WW) and water‐stress (WS) conditions.

Gene	Phene associations	Number of significant SNPs	Functional annotation
Zm00001d018394	Median metaxylem vessel area (WS and WW)	4	*DBPTF2*, DNA‐binding protein phosphatase 2C
Zm00001d028486	Maximum metaxylem vessel area (WS and WW), median metaxylem vessel area (WS and WW), volumetric flow rate (WS)	2	Chemocyanin
Zm00001d031332	Maximum metaxylem vessel area (WS and WW), median metaxylem vessel area (WS and WW)	4	Heat shock protein 90
Zm00001d038753	Maximum metaxylem vessel area (WS and WW), median metaxylem vessel area (WS and WW)	2	Ubiquitin domain containing 1
Zm00001d038754	Maximum metaxylem vessel area (WS and WW), median metaxylem vessel area (WS and WW)	2	ER lumen protein‐retaining receptor
Zm00001d045954	Maximum metaxylem vessel area (WS and WW), median metaxylem vessel area (WS)	1	RNA‐binding protein

Abbreviation: SNP, single nucleotide polymorphism.

### 3.4 Integration of root‐specific gene co‐expression network enables better prioritization of candidate genes

GWAS has been widely utilized as a tool to identify genes related to a particular phenotype, but it also presents many challenges. First, GWAS identifies many genes of which a notable proportion are false positives. Instead of relying solely on significance values associated with SNPs, we sought candidate genes with strong and consistent associations with root metaxylem phenes in maize across two field seasons. Second, phenotyping highly complex quantitative traits associated with small effect sizes remains a challenge in GWAS (Ingvarsson & Street, 2011; Schneider, Klein, Hanlon, Kaeppler, et al., 2020; Schneider, Klein, Hanlon, Nord, et al., 2020). Candidate gene selection and validation have been difficult when minute changes in phenotype are nearly imperceptible. Other work has proposed conducting transcriptome‐wide association studies (TWAS) to supplement GWAS for complex traits (Kremling et al., 2019), but no such root transcriptome data yet exist for the WiDiv. Instead, integrating GWAS with a gene co‐expression network enables candidate gene selection to be determined by network relationships inferring gene essentiality and biological function in its respective module (McDermott et al., 2009; Yu et al., 2004, 2007). Because these genes are more essential for maintaining gene‐gene interactions and signaling cascades, silencing hub and BN may carry a greater risk of inducing lethality. However, they also promise a higher likelihood of producing substantial modification in phenotype and function using other forms of mutation.

**FIGURE 3:**
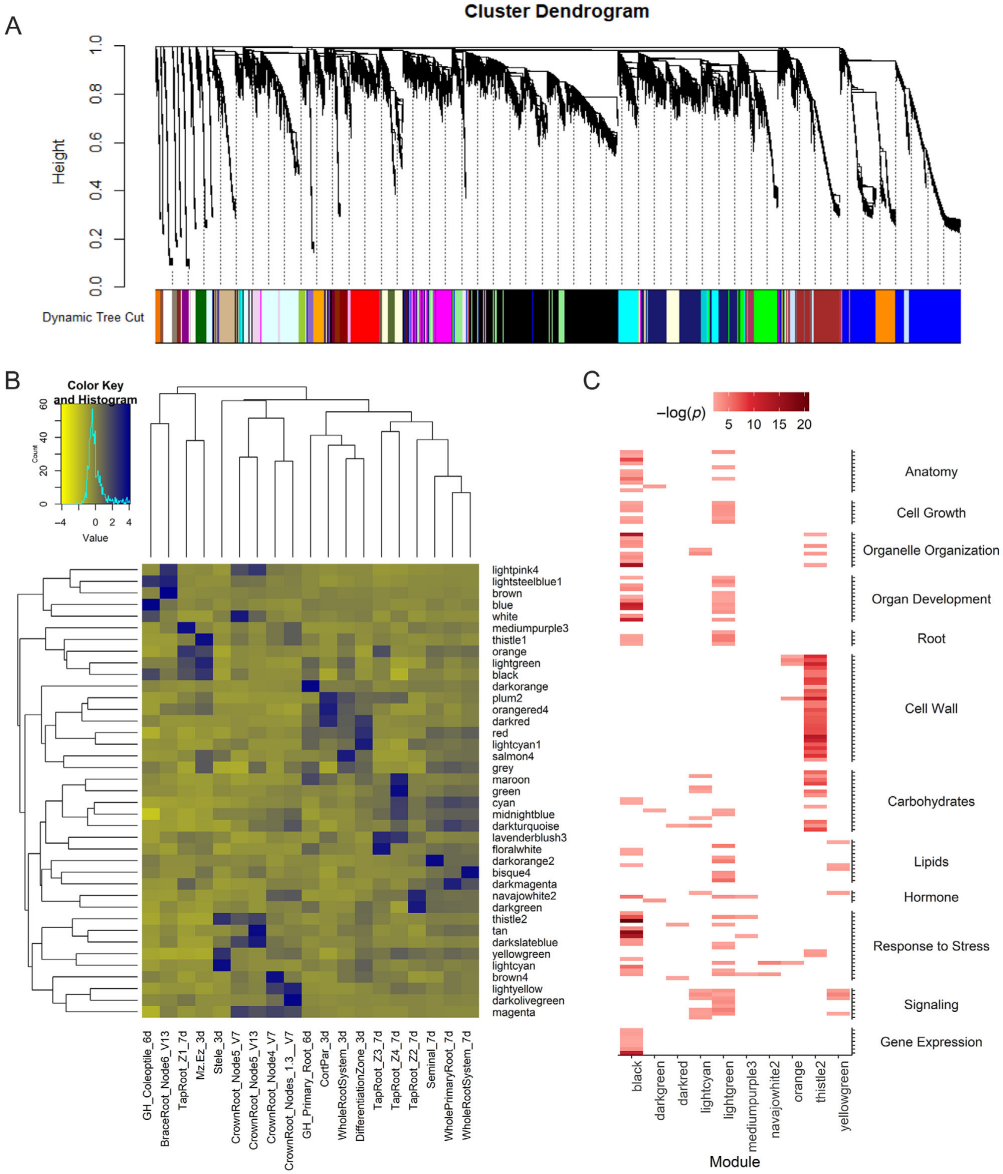
A gene co‐expression network and significant correlation of modules created with the Weight Gene Co‐expression Network Analysis (WGCNA) package. (A) Hierarchical clustering dendrogram displaying 39 modules of co‐expressed genes. (B) A heatmap showing the significance of the correlation [log(*p*)] between modules and various root tissues. (C) Inferred biological function of modules most likely associated with root metaxylem development based on gene ontology (GO) analysis conducted with agriGO. Only GO terms most relevant to xylem development are displayed. No GO terms were overrepresented by modules “lightcyan1,” “salmon4,” and “thistle1.”

To identify groups of genes with a high likelihood of regulating root xylem phenotypes, we constructed a gene co‐expression network using the expressed genes in 19 root tissues in B73 (Stelpflug et al., 2016) (Figure 3). Because this network was constructed using data from only one genotype, it does not take account for all genotypic variation in potential gene interactions that we may observe in this population. However, this gene expression atlas offers the specificity to examine expression patterns in the root tissues relevant to our phenes of interest. Using signed network analysis, we identified 39 modules of co‐expressed genes, each one labeled as a color (Figure 3A). The membership of each module ranged from 35 (module “lavenderblush3”) to 5987 (module “black”) genes. Of those, 14 modules had greater potential for being associated with metaxylem phenotypes because of the significant correlation between the module eigengene and root tissue samples relevant to xylem development (Figure 3B; Table S6). Root tissues most relevant to xylem development (−log(*p*) > 2) are the stele 3 DAS (modules “lightcyan,” “thistle2,” and “yellowgreen”), the differentiation zone 3 DAS (“darkred,” “lightcyan1,” and “red”), the meristematic and elongation zone 3 DAS (“black,” “lightgreen,” and “thistle1”), the whole root system 3 DAS (“salmon4”), zone 1 of the primary root 7 DAS (“mediumpurple3” and “orange”), and zone 2 of the primary root 7 DAS (“darkgreen” and “navajowhite2”). Gene ontology (GO) analysis showed that several biological functions related to xylem development were overrepresented in the xylem‐related modules (Figure 3C). Modules “black” and “lightgreen” were most strongly associated with root morphology and anatomical development, whereas module “thistle2” was particularly associated with the cell wall and carbohydrate biosynthesis. GO terms related to signaling were most strongly represented in modules “lightcyan,” “lightgreen,” and “yellowgreen.” While the “black” module was most strongly associated with stress responses, six additional modules were shown to be associated with stress responses as well. Three remaining modules were associated with xylem‐related tissues (“lightcyan1,” “thistle1,” and “salmon4”), but no overrepresented GO terms were characterized by these modules (not shown). Of the 244 significant gene candidates identified in the GWAS, 103 reside in gene co‐expression modules relevant to xylem development—57 under WW conditions and 50 under WS (Table 2).

Because of their contribution to xylem vessel wall development and reinforcement, genes involved in cell wall processes are natural targets for further investigation. Three candidate genes in xylem‐related co‐expression modules are associated with cell wall‐related processes and lignin biosynthesis according to their Mapman annotation: alpha‐l‐arabinofuranosidase/beta‐d‐xylosidase isoenzyme ARA‐I (Zm00001d009060) associated with cell wall degradation, pectinesterase (Zm00001d022567), and 4‐coumarate‐CoA ligase‐like 4 (Zm00001d027519) (Table S5). Four additional candidate genes were proposed to be associated with the biosynthesis and metabolism of cell‐wall polysaccharides (Okekeogbu et al., 2019) (Table S5): a pectate lyase (Zm00001d015328), a chitin elicitor‐binding protein (Zm00001d027533), a protein disulfide isomerase (Zm00001d046399), and a glucan endo‐1,3‐β‐glucosidase (Zm00001d047712). Interestingly, all candidate genes in this group were associated with root metaxylem size‐related phenes under WS. We did not find any significant associations with cell wall‐related genes that are well known to directly influence xylem development. For instance, cellulose synthase 4 (*CesA4*), a known positive regulator of xylem differentiation in *Arabidopsis* (Taylor et al., 2003), contained a SNP with a consistent effect on phenotype but was detected just below the *p*‐threshold (−log(*p*) = 3.02).

**FIGURE 4:**
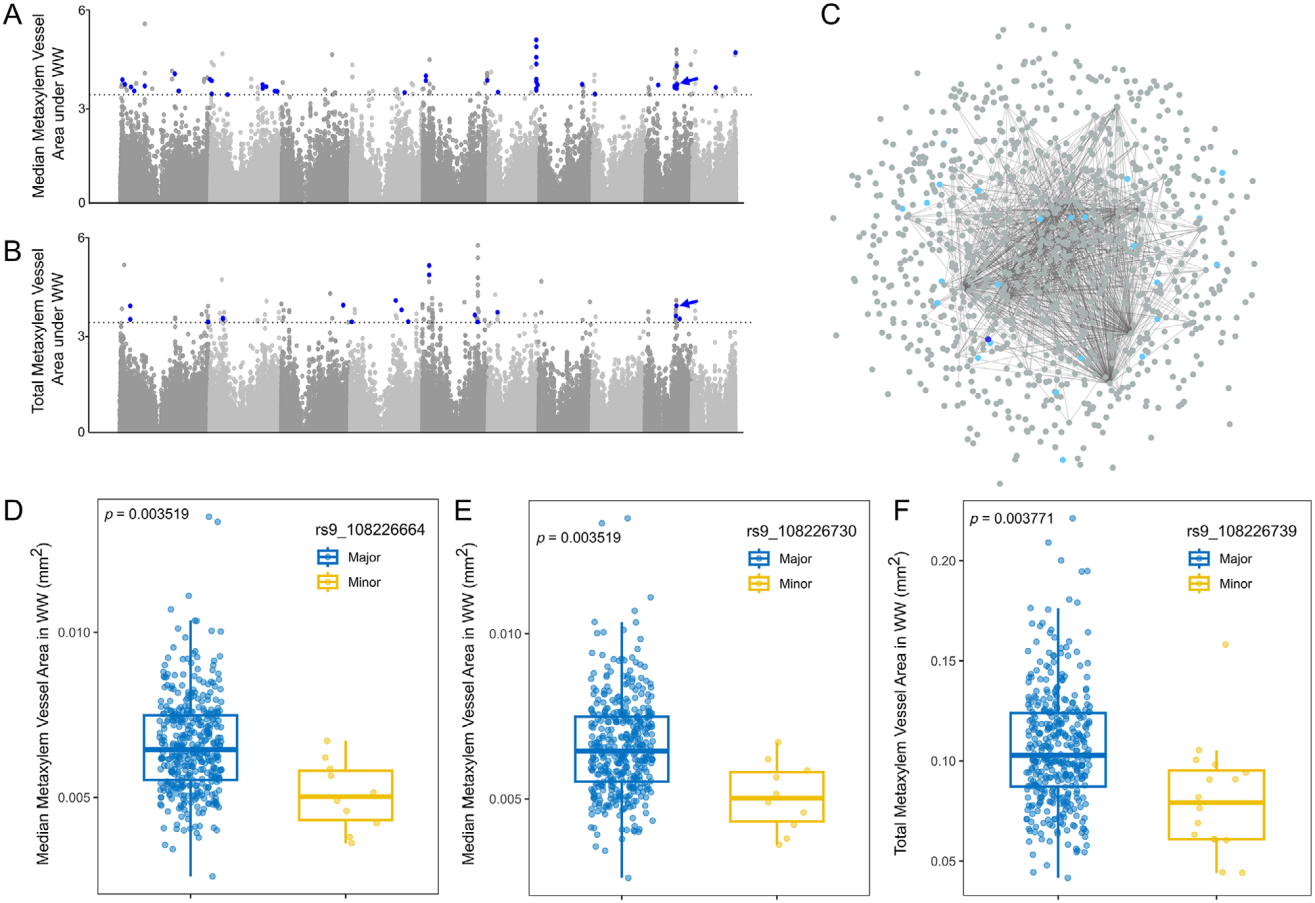
Results from the analytical pipeline integrating genome‐wide association study (GWAS) with a gene co‐expression network identified Zm00001d046838 as a strong candidate gene. Manhattan plots for (A) median metaxylem vessel area and (B) total metaxylem vessel area under WW (well watered). The blue arrows above chromosome 9 point to significant three significant single nucleotide polymorphisms (SNPs) located in the 5′‐UTR region of this candidate gene. (C) This gene lies within the “lightgreen” module, which was correlated with the meristematic and elongation zone 3 days after sowing (DAS). All nodes of the network have been illustrated where Zm00001d046838 is noted in dark blue, other candidate genes detected in the GWAS are labeled in light blue, and all other nodes are gray. Only first‐degree interactions mediated by Zm00001d046838 are displayed in the network graph. Significant reductions in median metaxylem vessel area under WW were observed in maize lines containing the minor allele for SNPs (D) rs9_108226664 and (E) rs9_108226730 (*p* = 0.003519). Similarly, a significant reduction in total metaxylem vessel area under WW in lines with the minor allele at SNP rs9_108226739 (*p* = 0.003771). Significant differences were determined by Welch's *t*‐test after testing for normality and equal variances.

Topology metrics of each module's network were used to infer the essentiality of each gene within the network (Table S7). HUB, or those that were most connected to the rest of the network, exhibit a high closeness centrality, while BN, those through which many connections are channeled, exhibit a high betweenness centrality. A gene may be both a bottleneck and a hub (BN/HUB). Within the network of each co‐expression module, genes in the 80th percentile and above of closeness centrality and betweenness centrality were classified as HUB and BN, respectively. Overall, of the 103 candidate genes identified through GWAS that belonged to gene co‐expression modules associated with root tissues most relevant to xylem development (Table S8), we prioritized 33 essential candidate genes: seven BN, nine HUB, and 17 BN/HUB (Table 3; Table S8). Of the GWAS candidate genes that reside in stele‐related modules (“lightcyan,” “thistle2,” and “yellowgreen”), three HUB, one BN, and three BN/HUB were identified (Table S8). Only one of these genes is annotated: ferritin homolog2 (*FER2*; Zm00001d023225), a hypothetical BN/HUB from “thistle2” associated with root metaxylem vessel number under WW conditions (Table S8). While the biological role of this gene candidate is not immediately clear, other candidates within the xylem‐related modules stand out. The pectinesterase (Zm00001d022567) discussed earlier was classified as a BN/HUB gene in the “salmon4” co‐expression module (Table S8). Zm00001d022295 encodes heat shock factor‐transcription factor 27 (*HSFTF27*), which was identified as a BN in the “black” module and is intriguingly associated with median metaxylem vessel area under WS. HSFTF27 has been shown in previous work to be more highly expressed in root tissues, responds to many environmental stresses like drought, and regulates primary metabolism (Haider et al., 2021; Wen et al., 2023). Zm00001d046838, which encodes a putative receptor‐like kinase family protein sharing a high degree of homology to HERCULES1 (*HERK1*), was associated with maximum and total metaxylem vessel area under WW (Figure 4). *HERK1* has been shown to be regulated by brassinosteroids and required for cell elongation in *Arabidopsis* (Guo, Li, et al., 2009; Guo, Ye, et al., 2009). Classified as a HUB in the “lightgreen” co‐expression module associated with the meristematic/elongation zone 3 DAS, three significant SNPs were detected near this gene located on chromosome 9 (Figure 4). These minor alleles associated with these SNPs are relatively rare—only 2%–4% of the population contained them—and their presence is associated with a 23% reduction in metaxylem vessel size‐related phenes under WW (Figure 4). Moreover, its rice ortholog (Os06g033430) was associated with median metaxylem vessel area (Fonta et al., 2023). Though these are not likely to be the causal SNPs as their minor alleles produce 5′‐UTR variants, this gene merits further investigation. Given that a reduction in metaxylem vessel diameter is demonstrably beneficial for improved drought tolerance in multiple species (Abd Allah et al., 2010; de Souza et al., 2013; Klein et al., 2020; Purushothaman et al., 2013; Richards & Passioura, 1989), this is a promising candidate to introduce to molecular breeding programs.

A gene candidate within the “mediumpurple3” module related to root zone 1 7 DAS—comprising the root tip, meristematic, and elongation zones—was associated with the volumetric flow rate under WW (Table S3). Δ(7)‐sterol‐C(5)‐desaturase (Zm00001d008569) is the rate‐limiting step in brassinolide biosynthesis and impairment of its activity is linked to defective longitudinal growth, irregularly spaced vascular bundles, and reduced xylem vessel size and number (Choe et al., 1999). Though not explicitly classified as a BN in our analysis, altering the activity of this gene would likely alter root metaxylem phenotypes as is evident by its relatively large minor allele effects (Table S5). This gene presents itself as an interesting candidate to better understand the biological mechanism underlying xylem development but would be a poor target for crop improvement. The minor allele is associated with an increase in volumetric flow rate under WW conditions, which would hamper efforts to breed maize lines that more efficiently conserve soil moisture by decreasing hydraulic conductance (Feng et al., 2016; Zaman‐Allah et al., 2011).

Interestingly, the gene encoding a nodulin‐like family protein (Zm00001d005082) contained 11 significant SNPs associated with maximum, median, and total metaxylem vessel areas and volumetric flow rate under WS and co‐expressed with genes highly expressed in the root meristematic/elongation zone 3 DAS (Table S5). This possible HUB lies within the genomic region on chromosome 2 that was densely populated with SNPs strongly associated with the total root metaxylem vessel area under WS (Table S6). Another nodulin protein (Zm00001d037160) contained one SNP strongly associated with metaxylem vessel number and co‐expressed with genes highly expressed in the stele 3 DAS (Table S5). Nodulins are not specific to legumes since homologs of nodulins have been found in several non‐nodulating species (Denancé et al., 2014). In non‐nodulating species, nodulin‐like proteins are transmembrane proteins shown to transport a wide range of compounds, and one has been shown to enhance root hair elongation in rice (Huang et al., 2013). Zm00001d005082 belongs to a nodulin‐like protein subfamily whose transport specificity has yet to be characterized in plants (Denancé et al., 2014). Our results suggest a potential novel role for nodulin and nodulin‐like proteins, which merits further investigation.

Since metaxylem anatomy has important implications for hydraulic function (Comas et al., 2013; Hacke & Sperry, 2001), the genetic architecture of root metaxylem phenotypes has important implications for plant performance under drought. Our findings suggest broadly that many genes influence root metaxylem phenotypes. In the classic paradigm, many of the candidate genes proposed here may be validated with the use of knockout or overexpression mutants to help elucidate the activity of these individual genes relative to root metaxylem phenotypes and subsequent performance outcomes under WS. However, while that strategy will certainly aid our fundamental understanding of vascular development in a crop species, it may not ultimately be helpful for producers. Our results indicate that identifying genetic variants advantageous in all environments may be difficult given the low degree of overlap between watering regimes (Table 3). Furthermore, given the knowledge gap in understanding shared genetic architecture between species, a gene with demonstrated utility in one plant species may not prove to be equally beneficial in another. Therefore, we must expand our understanding of the genetic mechanisms controlling vascular development into more model systems. The general workflow we developed in this study is one of many ways to tackle this issue, but it would require the generation of genetically diverse populations, adequate genetic resources, reliable phenotyping methods, and detailed gene expression atlases. Vascular development specific to cereal crops has been largely overlooked and must be better characterized to produce novel maize lines with hydraulic systems designed to safeguard yields under stress.

## AUTHOR CONTRIBUTIONS

**Stephanie P. Klein**: Formal analysis; investigation; methodology; visualization; writing—original draft. **Shawn M. Kaeppler**: Methodology; resources; writing—review and editing. **Kathleen M. Brown**: Supervision; writing—review and editing. **Jonathan P. Lynch**: Conceptualization; funding acquisition; methodology; project administration; supervision; writing—review and editing.

## CONFLICT OF INTEREST STATEMENT

The authors declare no conflicts of interest.

## Supporting information

Supplemental Table S1. Genotypes of the Wisconsin Diversity Panel grown in 2015 and 2016.
Supplemental Table S2. Mean values of root metaxylem phenes for the 95‐genotype subset of the Wisconsin Diversity Panel grown in 2015 under WW and WS conditions.
Supplemental Table S3. Raw data of root metaxylem phenes observed in the Wisconsin Diversity Panel grown in 2016 under WW and WS conditions.
Supplemental Table S4. Number of SNPs exhibiting consistent effects on root phenotypes in each treatment across both seasons.
Supplemental Table S5. Significant SNPs and candidate genes identified via GWAS for associated with all root metaxylem phenes under well‐watered and water stress conditions. MXAmax, maximum root metaxylem vessel area; MXAmed, median root metaxylem vessel area; MXAtot, total root metaxylem vessel area; MXN, metaxylem vessel number; VFR, volumetric flow rate.
Supplemental Table S6. List of 19 root tissues used to build the root‐specific gene co‐expression network.
Supplemental Table S7. Statistics describing network topology and membership size for each co‐expression module strongly associated with root tissues relevant to root metaxylem development.
Supplemental Table S8. Candidate genes that were also identified as genes essential to network function (i.e. bottlenecks, BN; bottleneck/hubs, BN/HUB; and hub, HUB) in xylem‐relevant modules. MXAmax, maximum root metaxylem vessel area; MXAmed, median root metaxylem vessel area; MXAtot, total root metaxylem vessel area; MXN, metaxylem vessel number; VFR, volumetric flow rate.

Supplemental Figure S1. Correlations between mean values of each genotype under well‐watered (WW) and water stressed (WS) conditions for each metaxylem phene in 2015 (black) and 2016 (grey). The solid black or grey lines indicate the slope of the correlation. The solid blue line indicates where x = y.
Supplemental Figure S2. Q‐Q plots assessing the fitness of the model for GWAS of root metaxylem phenes under well‐watered (WW) and water stress (WS) conditions.
Supplemental Figure S3. General outline of the filtering steps used to identify SNPs with consistent allelic effects in separate GWAS conducted with data collected in 2015 and 2015. (A) A substantial proportion of the SNP panel was included in each GWAS. Binned scatterplots showing (B) the full SNP panel and (C) the filtered set containing SNPs of consistent minor allele effects. The gradation of the bin corresponds to the number of SNPs residing in the bin where densely populated bins are light blue while more sparsely populated bins are dark blue.
Supplemental Figure S4. Mapman ontogenic categories for annotated gene models associated with significant SNPs in well‐watered and water stress conditions.

## Data Availability

The datasets generated during and/or analyzed during the current study are available in the GitHub repository: https://github.com/spklein/xylemGWAS. All data generated or analyzed during this study are also included in this published article (and its supplementary information files).
